# World Stroke Day — October 29, 2013

**Published:** 2013-10-25

Approximately 795,000 strokes occur each year in the United States. A leading cause of disability, stroke occurs among all age groups, including newborns, children, young adults, and older adults (1), and will affect one in six persons worldwide during their lifetimes (2–4). This year’s theme for World Stroke Day, October 29, is “Because I Care.” It emphasizes that stroke is preventable and the benefits of prevention should be extended to the entire family (4).

With timely care and support, most stroke survivors can recover and regain their former quality of life. Everyone should take the following actions to reduce their likelihood of having a stroke: 1) know your personal risk factors for stroke, including high blood pressure, diabetes, obesity, high blood cholesterol, atrial fibrillation, and a history of having a transient ischemic attack or previous stroke and control or manage these conditions by working with health-care providers; 2) engage in physical activity regularly; 3) maintain a healthy diet high in fruits and vegetables; 4) limit alcohol consumption; 5) avoid cigarette smoke (if you smoke, seek help to stop now); and 6) learn to recognize the warning signs of a stroke* (call 9-1-1 immediately if you think someone is having a stroke).

CDC addresses stroke prevention through state-based programs to prevent heart disease and stroke, through the Paul Coverdell National Acute Stroke Registry, and through many partnerships, including the Million Hearts Initiative. Additional information on stroke prevention is available from CDC at http://www.cdc.gov/stroke, and additional information about World Stroke Day is available at the World Stroke Organization website (4).

## Footnotes

* Sudden numbness or weakness of the face, arm, or leg, especially on one side of the body; sudden confusion, trouble speaking or understanding; sudden trouble seeing in one or both eyes; sudden trouble walking, dizziness, loss of balance or coordination; or sudden severe headache.
